# A Person-Centered Approach to Adolescent Nonsuicidal Self-Injury: Predictors and Correlates in a Community Sample

**DOI:** 10.1007/s10964-022-01628-y

**Published:** 2022-05-19

**Authors:** Melinda Reinhardt, Kenneth G. Rice, Barbara S. Durán, Gyöngyi Kökönyei

**Affiliations:** 1 Institute of Psychology, ELTE Eötvös Loránd University, Budapest, Hungary; 2 14th District Medical Center, Child and Adolescent Psychiatry, Budapest, Hungary; 3 Center for the Study of Stress, Trauma, and Resilience, Department of Counseling and Psychological Services, Georgia State University, Atlanta, GA USA; 4 SE-NAP2 Genetic Brain Imaging Migraine Research Group, Hungarian Academy of Sciences, Semmelweis University, Budapest, Hungary; 5 Department of Pharmacodynamics, Faculty of Pharmacy, Semmelweis University, Budapest, Hungary

**Keywords:** Nonsuicidal self-injury, Mental health, Perfectionism, Latent class analysis, Adolescents

## Abstract

Growing incidence of nonsuicidal self-injury (NSSI) and a lack of intensive examination of NSSI variability among adolescents justify identification of latent classes based on the endorsement of different NSSI behaviors. Latent class analysis was used to detect the heterogeneity of past month NSSI among 322 high school students (73.2% female). Two interpretable latent classes emerged. The Severe/Multimethod NSSI class (39%) engaged in almost all forms of NSSI with high intensity and motivated mainly for intrapersonal reasons. The results imply that compared to Mild/Moderate NSSI group (61%), the Severe class is at greater risk for poor mental health, which can exacerbate further NSSI acts. In school settings, identifying adolescents who are vulnerable for more severe NSSI can help to interrupt NSSI trajectories to emerging adulthood.

## Introduction

Poor mental health, negative self-views and self-critical perfectionism are integral predictors and maintaining factors of nonsuicidal self-injury (NSSI; Hooley & Franklin, 2018). Studying NSSI as an unhealthy coping strategy in youth populations is crucial to better understand its growing incidence and individual differences in adolescents’ mental health and adaptation. Nevertheless, exploring associations between NSSI, subjective well-being, and perfectionism is unduly neglected in adolescence studies. In addition, a person-centered framework and focus on adolescents who have recently engaged in NSSI can help us to better understand the heterogeneity of NSSI. Against that background, the current study focuses on emotional, psychological, and social well-being, and perfectionistic attitudes in NSSI subgroups among adolescents.

The umbrella term of NSSI covers intentional self-harm acts but without the will to die (International Society for the Study of Self-Injury [ISSS], 2018). The most common forms of NSSI are cutting, scratching, hitting or banging, and burning (Klonsky et al., 2014) with gender differences in their prevalence. Females tend to engage in cutting, carving, and scratching in greater proportion, whereas males are more likely to hit themselves (Barrocas et al., 2012). In the last decade, different surveys have revealed an upward trend in the prevalence of NSSI among community adolescents (Cipriano et al., 2017) and emerging adults (Wester et al., 2018). In recent findings, lifetime prevalence of more than 50% for at least one episode of NSSI is not uncommon among youth populations (Calvete et al., 2015). Throughout life, NSSI is the most common during adolescence in nonclinical populations (Swannell et al., 2014). The typical onset of NSSI falls between 12–14 years (Glenn & Klonsky, 2009). However, longitudinal studies on NSSI observed downward trends in point prevalence from mid to late adolescence (Plener et al., 2015).

NSSI acts strongly link to numerous unhealthy psychological processes (e.g., difficulties in emotion regulation, negative affectivity, alexithymia, self-criticism; McKenzie & Gross, 2014), impulsivity (Lockwood et al., 2017), and personality traits such as neuroticism (Allroggen et al., 2014) and perfectionism (Gyori et al., 2021a). NSSI behaviors can also occur in different psychiatric syndromes, primarily in mood, personality, anxiety, and substance use disorders (Nitkowski & Petermann, 2011). In parallel, life satisfaction and global well-being are patently lower in NSSI samples (Gyori et al., 2021b). A low level of subjective well-being is itself a risk factor for unhealthy emotion regulation (Verzeletti et al., 2016) and psychopathology (Grant et al., 2013). In addition, NSSI not only relates to mental disorders and may appear without mental illness symptoms, but poor mental health and unhealthy emotion regulation are closely tied with the risk of NSSI, irrespective of age and gender (Wolff et al., 2019).

Adolescence is one of the life periods that brings about the largest changes in biological, psychological, and social structures. Maturational changes have far-reaching effects on adolescents’ mental health, emotion regulation, and behavior, even risk-taking acts. Specifically, developmental changes in the stressor-sensitive brain regions and not fully matured prefrontal cortical regions can increase the emotional and psychosocial imbalance during this period (Spear, 2000). This age-dependent sensitivity has significant effects on self and emotional regulation, and on subjective well-being. Greater emotional instability and dysregulation could be linked to the onset of NSSI typically in early adolescence (Glenn & Klonsky, 2009) and, compared with other life stages, the highest point prevalence of NSSI during adolescence, specifically in middle adolescence (Brown & Plener, 2017). An integrated theoretical model of the development and maintenance of NSSI (Nock, 2009) suggests that NSSI emerges and continues because these kinds of behavior can promptly regulate unmanageable affective and social experiences. A set of distal (e.g., genetic predispositions to high emotional reactivity and familial criticism) and proximal risk factors (e.g., poor distress tolerance and high aversive emotions), as well as NSSI-specific vulnerability factors (e.g., high self-criticism or NSSI is a relatively easily accessible process for emotion regulation) can increase NSSI as a manifestation of unhealthy coping (Nock, 2010). It is important to underline that self-punishment and self-criticism are one of the most common motivations for NSSI (Nock & Prinstein, 2004). These specific “self-abusive” NSSI-vulnerability factors are essential parts of unhealthy perfectionism.

Beside poor mental health, one of the most important NSSI related variables is the transdiagnostic factor of perfectionism, often conceptualized as perfectionistic strivings (high performance expectations) and another factor referred to as perfectionistic concerns (excessive self-criticism, concerns about making mistakes, and perceived inadequacy in meeting expectations). More so than strivings, elevated perfectionistic concerns seems to be a significant risk and maintaining factor in self-injury (Limburg et al., 2017). However, relatively few research studies have tested the associations between these phenomena (Gyori et al., 2021a). The underlying common mechanism in NSSI and perfectionistic concerns could be the high level of self-punitiveness, self-criticism, and shame (Flett et al., 2012). On this basis, the perfectionistic concerns factor often is referred to as self-critical perfectionism (Dunkley & Blankstein, 2000). Moreover, unhealthy perfectionistic characteristics are typically associated with elevated negative emotions and poorer well-being (Fedewa et al., 2005). Because the main reason for NSSI is regulating negative affective states (Nock, 2009), it seems possible that NSSI can reduce the negative emotions emerging from increased self-criticism and perfectionistic concerns (Chester et al., 2015).

Most studies which analyze the associations between NSSI, mental health, and personality traits have been based on variable-centered approaches. A relatively small number of studies have used person-oriented approaches such as Latent Class Analysis (LCA) or Latent Profile Analysis (LPA) to identify distinct subgroups in NSSI. Furthermore, even fewer studies have implemented classification or mixture modeling approaches in studies of NSSI among adolescents. Although few in number, results based on person-oriented analyses have revealed important heterogeneity in NSSI.

Several studies have found support for four classes among young adults (Peterson et al., 2019). Although the labels have varied somewhat, the findings seem very consistent in identification of classes. Generally, one class (e.g., “mild/experimental NSSI”) featured low NSSI with low levels of both intrapersonal and interpersonal motivations engaging in NSSI and expressed the lowest levels of mental health problems (e.g., anxiety, depressive or personality disorder), as well as the lowest emotion regulation difficulties (Singhal et al., 2021). At the other extreme, members of a second class (e.g., “automatic functions/suicidal” or “severe NSSI”) had high and even multiple NSSI endorsement because of intrapersonal motivations and showed severe mental health symptoms (Klonsky & Olino, 2008). The two other intermediate groups (e.g., “multiple function/anxious NSSI” or “moderate NSSI” and “moderate multiple functions NSSI”) can be characterized by various combinations of moderate NSSI endorsement in different NSSI methods and diverse levels of psychological distress or internalizing symptoms (Case et al., 2020). There have been instances in which a fifth subgroup, labeled “multimethod” with the highest level of NSSI-versatility and psychopathology, has emerged (Bracken-Minor et al., 2012). In each of these LCA studies, the largest number of participants was concentrated in the “experimental NSSI” or “low NSSI” subgroups (between 21% and 73%), whereas the smallest concentrations occurred in the “multiple functions/anxious” or the “high/severe NSSI” subgroups (approximately 10% of who engaged in self-injury).

Two studies could only be located that analyzed NSSI latent classes among adolescents. In a Turkish youth sample, four subtypes of NSSI emerged: (1) low endorsement in NSSI acts, (2) high endorsement in self-hitting, (3) high rates of self-cutting, and (4) high rates of multiple forms of NSSI (Somer et al., 2015). The multiple NSSI class showed significant psychological vulnerability. Among justice-involved juveniles, “low NSSI,” “moderate NSSI,” and “high NSSI” classes relating to different forms of NSSI were detected (Reinhardt et al., 2021). The “low NSSI” group had the fewest dissociative experiences. Comparable NSSI subtypes appear in developmental research. In a 3-year longitudinal framework, three developmental trajectories in NSSI engagement were detected among middle school students: (1) stable low self-injury, (2) increase in initial low self-injury, and (3) decrease in initial moderate level of self-injury (Huang et al., 2021). In contrast, over a 1-year time frame, four NSSI subgroups were distinguished among adolescents: (1) no or marginal NSSI, (2) experimental (initially low and continuously decreasing NSSI level), (3) moderate decreasing (decreasing level from a moderate initial stage), and (4) high fluctuating (oscillating but persistently high NSSI level; Wang et al., 2017).

## Current Study

Only a few studies have attempted to map the distinct patterns and multiple dimensions of NSSI behaviors among adolescents, despite the typical onset of NSSI in early adolescence and the fact that NSSI is the most frequent during adolescence in nonclinical samples. Furthermore, in adolescence, an increase in risk behaviors is often observable. Accordingly, the primary aim of this study was to identify latent classes based on the endorsement of different self-injury methods in a Central-European juvenile sample. This person-centered approach to risk classification allowed us to examine the diversity in NSSI. Other novelties in this study include the age range and representation of the youth from middle to late adolescence in the general community, and the focus only on juveniles who have engaged in NSSI in the past month. The results have the potential to be relevant for identifying adolescents who are at more immediate risk for NSSI. In addition, predictors (i.e., age, gender, mental health, perfectionism, and motives for NSSI) and correlates of latent risk patterns (e.g., experienced pain during NSSI or time urgency of self-injury intent) were examined. Grounded in past research, it was hypothesized that emergent classes would vary according to different severity indicators such as frequency and versatility. Finally, it was also hypothesized that the most severe NSSI class would be predicted by significant intrapersonal motivations for self-injury and higher levels of perfectionistic concerns and would have the poorest subjective well-being compared with other classes.

## Method

### Participants and Procedure

The sample size was informed by guidance from several sources. Latent class analysis is essentially a descriptive and exploratory approach used for categorical indicator data that is aimed at determining whether latent classes exist and, if so, evaluating the number of classes supported by the data. It was stated that, “if the goal is a purely descriptive analysis where the researcher simply seeks to describe sample characteristics, there is often no minimum requirement beyond the basic requirements for computation of a solution” (Woo et al., 2018, pp.831–832). Alternatively, several authors have conducted simulation studies to provide guidance for sample size and power estimates in latent class analyses. The number of classes, change in information indices or likelihood ratio tests, and the extent of class separation are important considerations in several of the simulation models presented in previous research. In advance, the degree of class separation was unknown, as were the number of classes that would emerge in the current sample, however up to four classes have been observed in past studies. Assuming separation of these classes might be moderate to large, simulation studies suggest a sample size of 300 to 500 subjects should be sufficient to detect three or four classes that were moderately separated (e.g., Dziak et al., 2014 or Gudicha et al., 2017).

A total of 1232 14-to-20-year-old adolescents across 14 Hungarian secondary schools were invited to participate in a cross-sectional study with a focus on mental health, emotion regulation, perfectionism, and NSSI. A total of 173 students declined to participate or were absent during data collection, and another 44 students were excluded from the data analysis because of more than five missing responses per scale. A final sample of 1015 Hungarian adolescents (*M*_age_ = 16.81 years, *SD* = 1.42; 66.1% females) completed the questionnaires in their classrooms or computer rooms under the supervision of trained researchers. No teaching staff members were present. Participation in the study was voluntary and anonymous. Written informed consent was sought from all respondents and one of their parents. The study was approved by the Institutional Review Board of ELTE Eötvös Loránd University Faculty of Education and Psychology, and the work was conducted in accordance with the Declaration of Helsinki (World Health Organization WHO (2001)). Analyses were limited to the 322 adolescents (31.7% of the whole sample) who identified as engaging in at least one episode of NSSI in the past month. The mean age of this subsample was 16.72 years (*SD* = 1.41), with an age range between 14 and 20. Most were female (73.2%; *n* = 235; 26.8% were male, *n* = 86), and one respondent did not disclose gender. Regarding grade, 30.1% (*n* = 97) attended 9th grade, 22.3% (*n* = 71) attended 10th grade, 26.7% (*n* = 85) attended 11th grade, and 20.4% (*n* = 65) attended 12th grade in the Hungarian high school system.

### Measures

#### Nonsuicidal self-injury

NSSI was measured with the Inventory of Statements about Self-Injury (ISAS; Klonsky & Glenn, 2009). The first part of the ISAS assesses the lifetime frequency of 12 self-injury methods (e.g., cutting, severe scratching, hitting, biting, burning; see Table 3 for the full list of methods). Participants were asked to estimate the number of times they have intentionally engaged in each of the self-injury methods. Consistent with Weller et al., (2020) and our interest in identifying different risk classes, participant item responses were dichotomized into 0 (never engaged in the method) or 1 (engaged in the method at least one time) (see also Masyn, 2013). The 12 descriptive binary items of NSSI methods were used as indicators of latent classes.

Follow-up ISAS items regarding NSSI are completed only by those who engaged in at least one of the methods at least one time. Four such items detect additional aspects of NSSI behaviors (experienced pain during NSSI, alone during self-injury, urgency of the NSSI episode, desire to stop NSSI). Those four items were used as criterion variables or potential correlates of any emergent latent classes. Two further questions refer to the first and the last engagement in NSSI.

The short version of the second part of the ISAS contained 26 items to measure possible motivations or functions for self-injury (Washburn et al., 2012). Each item is rated 0 (*Not relevant*), 1 (*Somewhat relevant*), or 2 (*Very relevant*) as a motivation. The motives can be represented as two broader intrapersonal and interpersonal factors (Klonsky & Glenn, 2009). The intrapersonal functionality of NSSI is measured by 10 items and covers self-regulation aspects (i.e., affect regulation, anti-dissociation, anti-suicide, marking distress, and self-punishment). Interpersonal motives are measured by 16 items that reflect the social rationales for self-injury (i.e., autonomy, interpersonal boundaries, interpersonal influence, peer-bonding, revenge, self-care, sensation seeking, and toughness). Acceptable internal consistency was observed for both intrapersonal (*α* = 0.80), and interpersonal factors (*α* = 0.88) in the original long form of the second part of the ISAS (Klonsky & Glenn, 2009). Our results were similar in terms of McDonald’s omega (*ω* = 0.78 for the intrapersonal and *ω* = 0.80 for the interpersonal factor). These two motives, along with other demographic (age, gender) and psychological variables (perfectionism, mental health), were used as predictors of latent classes.

#### Perfectionism

The 8-item Short Almost Perfect Scale (SAPS; Rice et al., 2014) specifies dispositional perfectionism represented by two subscales. The 4-item Standards subscale assesses perfectionistic strivings with emphasis on personal performance expectations. The 4-item Discrepancy subscale measures self-critical perfectionistic concerns, specifically about the perceived difference between personal expectations and performance in meeting those expectations. Each item is rated on a 7-point scale ranging from 1 (*Strongly disagree*) to 7 (*Strongly agree*). In this study, item responses are averaged such that scores could range from 1 to 7. Good reliability coefficients were obtained for both subscales in the original (Standards *α* = 0.85 and Discrepancy *α* = 0.87) (Rice et al., 2014), as well as in the current study (Standards *ω* = 0.88 and Discrepancy *ω* = 0.77).

#### Subjective well-being

The Adolescent Mental Health Continuum – Short Form (A-MHC-SF; Keyes, 2006) is a 14 item-long self-reported questionnaire that provides a global well-being index as well as three well-being facet scores: (1) emotional (3 items), (2) psychological (6 items), and (3) social well-being (5 items). Adolescents assess the frequency of positive mental health experiences in the past month from *Never* (0) to *Every day* (5). In an adolescent sample, good score reliability results were reported for the general scale (*ω* = 0.91), as well as the three subscales (*ω* = 0.89, 0.84, and 0.82 for emotional, psychological, and social well-being, respectively; Rogoza et al., 2018). McDonald’s *ω* coefficients were also good based on scores in the current study (*ω* = 0.90, 0.83, 0.84, and 0.72 for global, emotional, psychological, and social well-being, respectively). Because of prior results (Rogoza et al., 2018) and high correlations between the three well-being subscales in the current study, the ordered categorical score described by Keyes (2002) was used to estimate broad mental health of the subjects. Specifically, Keyes (2002) recommended respondents could receive one of three mental health ordinal ratings based on the patterns of their A-MHC-SF scores: 0 = languishing persons are characterized by very low positive mental health states on the three (emotional, psychological, and social) well-being domains; 1 = adolescents with moderate mental health experience average rates on the three subjective well-being domains, and 2 = flourishing individuals have high subjective well-being on all domains. This approach is appealing in large part because it presents a practically meaningful and easy to interpret mental health or well-being index.

### Data Analysis

IBM SPSS Version 27 and M*plus* Version 8.7 (Muthén & Muthén, 1998–2021) were used in analyses. The M*plus* default of full information maximum likelihood addressed missingness and generated unbiased estimates of parameters. The robust MLR estimator was used, which also is the default in M*plus* for latent class analyses (LCAs).

#### Latent class analysis

LCA was conducted to identify subgroups of participants based on their NSSI involvement as reflected in their responses to the 12 ISAS items. The class enumeration process in LCA involves a series of alternative models in which incrementally different numbers of classes are compared. In each model, thresholds for the categorical ISAS indicators were allowed to be freely estimated between classes. Each model was tested with 5000 starts, 200 final stage optimizations, and 100 iterations.

There are statistical and practical considerations in determining the best model from a set of alternative models. Model testing begins with a k = 1 (single class) model, then advances through alternative models with each estimating an additional possible class. This class enumeration process is evaluated based on several information indices, including the Consistent Akaike Information Criterion (CAIC), Bayesian Information Criterion (BIC), and sample-adjusted BIC (SABIC), with smaller values corresponding to better-fitting models. The Lo-Mendell-Rubin (LMR) likelihood ratio test (LRT) and the parametric bootstrap (BLRT; McLachlan & Peel, 2000) also were used to compare k-class with k-1 class models. Significant LMR or BLRT effects indicate that a current k-class model is statistically better than the preceding k-1 class model. Large samples might not produce a clear minimum point for the information indices and may not reveal nonsignificant LRT estimates (Marsh et al., 2009). In those instances, plots of the CAIC, BIC, and SABIC can be used to locate a potential point, at which adding more classes does not substantially change the indices (Morin & Marsh, 2015). Practical considerations are also an important part of the class enumeration process and evaluation of alternative models. Such considerations include the interpretability of a class solution and whether there is a consequential proportion of people populating a class. These practical considerations can be especially helpful in reconciling the not uncommon situation of information indices and LRT results being inconsistent with one another. Classification accuracy is less important in class enumeration but informative for interpretation. The relative entropy index (typically 0.80 or larger) would be consistent with clearer class differentiation.

## Results

### Preliminary Analyses

Descriptive statistics and correlations between predictors and outcome variables are shown in Table 1. The correlation between Standards and Discrepancy was medium-strong, unlike results previously reported for those perfectionism factors (e.g., Rice et al., 2014). Although correlated with each other, the two factors related differently to positive mental health subscales. Standards was ostensibly uncorrelated with positive mental health whereas Discrepancy was inversely and moderately associated with positive mental health. Higher Standards and Discrepancy weakly associated with higher experienced pain and aloneness during self-harm. Adolescents who have higher Standards are more likely urged to engaging is NSSI, and those who have higher Discrepancy are more likely to engage in NSSI due to intrapersonal motives.

**Table 1:** Descriptive statistics and correlations between predictors and correlates (outcomes)

Variable	M (SD)	1	2	3	4	5	6	7	8	9	10	11	12	13	14	15
1 Age	16.73 (1.44)	**--**														
2 Gender		0.05	–													
3 Standards	5.15 (1.38)	0.09	−0.25	0.88												
4 Discrepancy	4.53 (1.38)	0.04	−0.25	0.51	0.77											
5 Emotional Well-being	8.32 (3.39)	−0.14	0.21	−0.08	−0.45	0.83										
6 Psychological well-being	17.02 (6.73)	−0.08	0.16	0.06	−0.37	0.71	0.84									
7 Social Well-being	9.87 (4.95)	−0.14	0.14	−0.03	−0.27	0.66	0.70	0.72								
8 Global Well-being	35.21 (13.46)	−0.12	0.19	0.00	−0.40	0.85	0.93	0.88	0.90							
9 Mental health categories		−0.06	0.14	0.03	−0.35	0.74	0.82	0.70	0.85	–						
10 Pain		−0.01	0.06	−0.13	−0.17	0.09	0.03	0.00	0.04	0.02	–					
11 Alone		−0.02	0.19	−0.15	−0.18	0.22	0.21	0.20	0.24	0.19	0.27	–				
12 Urgency		−0.18	0.00	−0.14	−0.05	0.09	0.12	0.13	0.13	0.10	0.08	0.12	–			
13 Stop		0.11	0.14	−0.05	0.05	−0.17	−0.13	−0.19	−0.18	−0.17	−0.02	0.10	−0.04	–		
14 Intrapersonal motives	7.47 (5.77)	0.04	−0.18	0.10	0.22	−0.25	−0.24	−0.22	−0.27	−0.19	−0.26	−0.20	−0.25	0.21	0.78	
15 Interpersonal motives	5.76 (5.87)	−0.10	0.00	0.00	0.09	−0.07	0.00	0.02	−0.01	−0.04	−0.10	0.08	0.00	0.17	0.38	0.80

Results derived from multiple imputation pooled data, *N* = 322. Underlined diagonal values represent Omega (*ω*) reliability coefficients. Gender was coded 0 = girls and 1 = boys. Mental Health categories were coded 0 = Languishing, 1 = Moderate, 2 = Flourishing. Pain (experienced pain during self-injury), and Alone (adolescent who engaged in self-injury was alone during the act or not) were coded 1 = yes, 2 = sometimes, 3 = no, and Stop (the desire to stop engaging in self-injury) was coded 1 = yes and 2 = no. Urgency (the typical amount of elapsed time between the urge to self-injury and acting on the urge) was coded 1 = less than 1 h, 2 = 1 to 24 h, and 3 = over 24 h. Pearson correlations were calculated for continuous scores, point-biserial correlations were calculated for correlations involving dichotomous variables, and Spearman rho correlations were calculated for correlations involving Mental Health, Pain, Alone, and Urgency.

|*r*| > 0.10, *p* < 0.05 and |*r*| > 0.17, *p* < 0.001, two-tailed test.

Moreover, adolescents who reported they are alone during self-harm are more likely to experience lower subjective well-being, higher pain during self-harm, and more explicit urge to self-harm. Adolescents who have increased urge to engaging in self-harm have lower psychological, social, and global well-being, as well as higher Standards. Thus, adolescents who want to stop self-harm can be identified by higher subjective well-being.

Higher intrapersonal motivation behind self-harm is associated with higher Discrepancy, lower positive mental health, more pain and aloneness during self-harm, as well as higher urge for and commitment to self-harm. Interpersonal motivation correlated positively and moderately with intrapersonal motivation, and weakly with the commitment to self-harm.

Age only correlated with well-being factors and NSSI impulsivity, and commitment. Younger adolescents were more likely to be characterized by higher emotional, social, and global well-being, as well as longer amount of elapsed time between the urge to self-harm and acting on the urge. Moreover, younger adolescents tended to desire stopping NSSI behaviors more frequently than older adolescents. Girls showed lower positive mental health, and higher Standards and Discrepancy than boys. In addition, compared to boys, girls were more likely to report being alone during self-harm, wanting to stop self-harm, and engaging in self-harm motivated by intrapersonal reasons.

### Latent Class Analyses

Fit and classification results appear in Table 2. The LCA based on the 12 categorical items showed a decline in the BIC and CAIC values for the two-class model and then both increased thereafter. The SABIC declined for all the models tested. The significant LMR suggested support for the two-class model, whereas the BLRT results supported the four-class model. Based on the combination of results, the clearest and most consistent support across several indices was obtained for the two-class model. That model also had no estimation issues; the four-class model suggested by the BLRT results converged but only after logit thresholds for three of the indicators were fixed to stabilize optimization. The two-class model provided reasonable class sizes and the entropy estimate suggested good classification separation. Based on posterior probability estimates, Class 1 represented approximately 39% of the sample (*n* = 125) and Class 2 represented the remaining 61% of the sample (*n* = 197).

**Table 2:** Fit and classification accuracy results from latent class analyses

Model	k	#fp	LL	CAIC	BIC	SABIC	Entropy	LMR *p*	BLRT *p*
One class	1	12	−2262.29	4605.87	4593.87	4555.81			
**Two classes**	**2**	**25**	−**2057.75**	**4284.86**	**4259.86**	**4180.56**	**0.792**	**<0.0001**	**<0.0001**
Three classes	3	38	−2022.10	4301.63	4263.63	4143.10	0.759	0.122	<0.0001
Four classes	4	51	−2002.24	4349.99	4298.99	4137.23	0.812	0.777	0.02
Five classes	5	64	−1983.99	4401.55	4337.55	4134.55	0.778	0.107	0.30

Bolded row represents the candidate model. *#fp* Number of free parameters, *LL* Model log likelihood, *CAIC* Consistent akaike information criterion, *BIC* Bayesian information criterion, *SABIC* Sample-size adjusted BIC, *LMR* Lo, Mendell, and Rubin likelihood ratio test, *BLRT* Bootstrap likelihood ratio test.

**Fig. 1:**
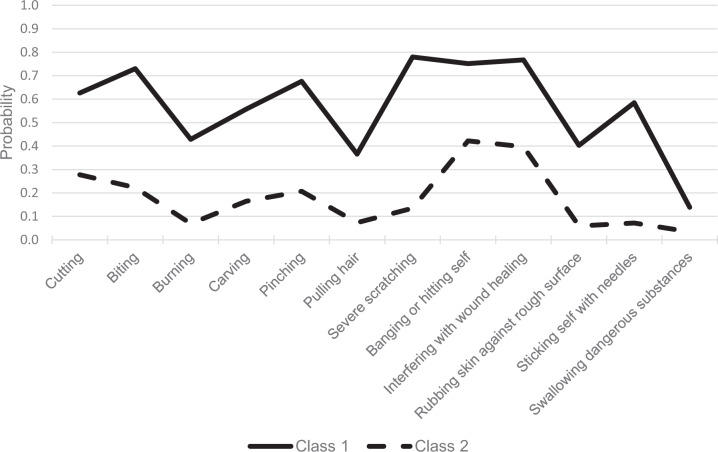
Probabilities of NSSI item endorsements based on the two-class model

Table 3 presents the conditional probabilities for the 12 ISAS items for the two-class model. The columns display the probabilities of “yes” responses to the item within each of the classes. To facilitate interpretation, probabilities are also displayed in Fig. 1. The results show a very different pattern of results that emerged for adolescents reporting they had recently engaged in self-harm behaviors. Most striking is Class 1, with a high likelihood of reporting 8 of the 12 NSSI behaviors. Several of those behaviors represented high risk for severe tissue-damage (e.g., cutting, biting, sticking self with needles) or other form of injury (e.g., banging or hitting self). Class 2 represented relatively lower risk with comparatively lower probabilities for severe self-injury with minimal likelihood of engaging in a few behaviors (mostly banging or hitting self and interfering with wound healing). Although most of the behaviors were clearly differentiated between the two classes, it was important to note that the probability of swallowing dangerous substances was not substantially different between the two groups.

**Table 3:** Conditional probabilities of item endorsements for the two-class LCA model

Item	Class 1	Class 2
Cutting	0.626	0.278
Biting	0.730	0.223
Burning	0.429	0.069
Carving	0.557	0.165
Pinching	0.676	0.207
Pulling hair	0.366	0.073
Severe scratching	0.780	0.135
Banging or hitting self	0.752	0.422
Interfering with wound healing	0.767	0.397
Rubbing skin against rough surface	0.403	0.059
Sticking self with needles	0.585	0.072
Swallowing dangerous substances	0.139	0.034

1 = highest probability of responding “yes” and 0 = lowest probability of responding “yes” (i.e., likely “no” response).

Local independence is an important assumption of LCA. Local independence “implies that latent class membership explains *all* of the associations among the observed items” (Masyn, 2013, p. 558). Local independence was evaluated by examining standardized bivariate residuals for each combination of items and response options in the two-class model. None of the standardized residuals exceeded |1.96 | (the largest was −1.93). Thus, the local independence assumption was tenable for the two-class model.

#### Predictors of class membership

The seven hypothesized predictors of latent classes were evaluated using the R3STEP auxiliary analysis procedure in M*plus*. That procedure produces multinomial logistic regression analyses in which classes are regressed on the covariates such that the estimated predictor effects for one class are evaluated in comparison to a reference class. Partial coefficients are derived in which the effects of one predictor are estimated while holding effects of other predictors constant. Regression coefficients for the various class comparisons are reported in Table 4. Missingness was relatively minimal for five of the covariates. There were no missing values for A-MHC-SF status, Standards, or Discrepancy, and only 1 missing value for gender (0.3%), and 4 missing values for age (1.2%). The major source of missingness for the predictors was attributable to the two motives (Intrapersonal and Interpersonal) with approximately 7.5% missing scores on those factors (*N* = 24). Because the R3STEP procedure uses listwise deletion when evaluating predictors of latent classes, multiple imputation was used in M*plus* to generate plausible values for missing data with those two variables (Rubin, 1987). Thirty imputation datasets were created based on available data for analyses. These data were aggregated using default features in the M*plus* program for proper adjustments of standard errors. Thus, results were pooled from the separate analyses of each dataset to arrive at the final set of reported findings.

**Table 4:** Multinomial logistic regression results for predicting class membership

Reference/Comparison	Predictor	Estimate	SE	*p*
Class 2/Class 1	Gender	−0.33	0.38	0.391
	Age	−0.12	0.11	0.288
	Standards	0.01	0.03	0.791
	Discrepancy	0.03	0.04	0.336
	Mental health	−0.70	0.26	0.007
	Intrapersonal motives	0.13	0.04	0.002
	Interpersonal motives	0.03	0.03	0.322

The R3STEP procedure in M*plus* is based on listwise deletion of missing data. Therefore, multiple imputation was used to impute values for the Motives factors resulting in *N* = 317 for these analyses. Gender was coded 0 = girls and 1 = boys. Mental Health was based on the ordinal scoring of the MHC-SF in which 0 = “languishing”, 1 = “moderate”, and 2 = “flourishing”.

As shown in Table 4, gender, age, perfectionism, and interpersonal motives did not differentially predict membership in either of the two classes. Compared to Class 2, Class 1 was more likely to report a lower level of mental health status, and a higher degree of intrapersonal motivation for self-injury. The most likely class designation was exported to evaluate the distribution of mental health categorization across the two classes. Approximately the same proportions of youth in Class 1 (56%) and Class 2 (57.3%) were in the “moderate” mental health status. The significant effect of mental health status predicting NSSI class could be attributable to differences at the low and high end of mental health. Approximately 32% of the youth in Class 1 were in the “languishing” status, whereas only 12.1% were in the “flourishing” status. In contrast, 16% of Class 2 youth were in “languishing” and 26.7% were in the “flourishing” status. Thus, there was comparatively greater risk of poor mental health for Class 1 compared with Class 2 and greater relative likelihood of better well-being for youth in Class 2 compared with those in Class 1. The only other variable to differentially predict membership in the classes was Intrapersonal Motives. Again, using the exported most likely class membership indicator, there was a medium to large effect size difference between the average Intrapersonal Motives score for Class 1 (9.15, *SD* = 4.01) and Class 2 (7.27, *SD* = 4.26), d = 0.69. Youth in Class 1 were substantially more likely to endorse intrapersonal reasons for engaging in self-harm compared with youth in Class 2.

#### Correlates of class membership

Each of the correlates were binary or ordered categorical variables so the DCAT procedure in M*plus* was used to evaluate differences between the two classes. Similar to R3STEP, the M*plus* DCAT procedure is conducted after listwise deletion of missing data. At most, 2.5% (*n* = 8) of the participants had missing data on one of the correlates. Nevertheless, these analyses were based on data from 30 multiple imputation datasets resulting in a final sample size of 322. There were no substantial differences in parameter estimates based on analyses using multiple imputation datasets compared to results based on the original data (maximum difference in a probability estimate was −0.004). When original data are analyzed, the results provide a separate, overall chi-square test for the association between the classes and each outcome. Those tests are not available when results are based on multiple imputation datasets. Therefore, results based on the original data were reported.

The omnibus test revealed a significant association between classes and experienced pain during NSSI (χ^2^ [2, *N* = 319] = 7.64, *p* = 0.022) and urgency of the NSSI episode (χ^2^ [2, *N* = 316] = 14.56, *p* = 0.001). In contrast, there was not a significant association between the classes and being alone during self-harm (χ^2^ [2, *N* = 318] = 2.86, *p* = 0.239) or between the classes and the desire to stop NSSI (χ^2^ [2, *N* = 314] = 3.53, *p* = 0.060). Class probabilities for these comparisons are displayed in Table 5.

**Table 5:** Probabilities for associations between class membership and psychological correlates

Correlate (Outcome)	Response	Class 1	Class 2	χ^2^ (2)	*p*
Do you experience physical pain during self-harm?	Yes	0.369	0.324	7.64	0.022
	Sometimes	0.466	0.353		
	No	0.165	0.323		
When you self-harm, are you alone?	Yes	0.668	0.566	2.86	0.239
	Sometimes	0.221	0.245		
	No	0.110	0.188		
Typically, how much time elapses from the time you have the urge to self-harm until you act on the urge?	<1 h	0.414	0.435	14.56	0.001
	1 to 24 h	0.231	0.062		
	>24 h	0.355	0.503		
Do/did you want to stop self-harming?	Yes	0.743	0.845	3.53	0.060
	No	0.257	0.155		

*N* ranged from 314 to 319.

The differences between the two classes were consistent with Class 1 being more at-risk than Class 2. For example, in terms of pain experienced during NSSI, compared to Class 2, Class 1 was more likely to respond with “yes” or “sometimes”. In terms of the time lapse between urgency to engage in NSSI, about two-thirds of Class 1 youth were more likely to act on the urge within 24 h, whereas about half of the Class 2 youth were likely to act within 24 h. The two classes were comparable in their likelihood of being alone when engaging in NSSI; approximately 80–90% of the youth responded “yes” or “sometimes” to that item. Likewise, the desire to stop NSSI did not significantly differentiate the two classes. About 74% of those in Class 1 and 85% of those in Class 2 reported that they wanted to stop self-harming.

## Discussion

Despite the significantly growing incidence of NSSI in adolescent populations, research focused on exploring the diversity of such behaviors among high school-age students is lacking. Using Latent Class Analysis, this study aimed to identify and map the characteristics of adolescent groups who reported self-injurious acts in the previous month. This person-oriented solution can separate juveniles into classes based on their self-evaluation on the severity of different NSSI methods.

Two interpretable classes emerged that are partly aligned with previous NSSI LCA studies. However, by the same token, it is important to emphasize that in previous studies, lifetime or past year prevalence of NSSI was usually used in the analyses. In the present study, current (past month) NSSI acts were considered during class identification. This is particularly crucial for youth populations, based on the potential of rapid and extensive physical and psychological changes during this life stage (Hazen et al., 2008).

According to the results, 39% of the adolescents who had engaged in NSSI in the past month reported a severe engagement in multitude of NSSI behaviors such as cutting, biting, carving, pinching, severe scratching, hitting, interfering with wound healing, and sticking self with needles, together with moderate endorsement in burning, pulling hair and rubbing skin against rough surface. The adolescents in this subgroup engaged seriously and persistently in almost all the assessed NSSI methods. In view of this severity, this latent class was labeled as “Severe/ Multimethod NSSI.” Members of this group expressed more intrapersonal motivations underlying NSSI, reported more frequent physical pain during self-harm, and experienced a stronger urge for self-harm acts. More simply, NSSI may provide this group an easily available self-regulating tool against burdensome emotions and cognitions (Klonsky & Glenn, 2009). This reflects contemporary theories of NSSI, which contextualize NSSI using an affect regulation framework (Chapman et al., 2006), as well as theories which explain NSSI from a developmental perspective. In a challenging life period like adolescence, NSSI is immediately available when affective and social experiences overwhelm the emotion regulation capacity of the young people (Nock, 2009).

Furthermore, higher intrapersonal motives underlying NSSI are significantly associated with higher perfectionistic concerns in this study. It may follow that any of these demanding affective states are arising from overwhelming self-criticism and perfectionistic concerns (Chester et al., 2015). This is confirmed by the developmental model of NSSI, in which high self-criticism is an NSSI-specific vulnerability factor for NSSI (Nock, 2010). Self-criticism is a key element in unhealthy perfectionism (Dunkley and Blankstein, 2000), which strongly links to diminished well-being (Fedewa et al., 2005). Therefore, there are reasonable grounds for believing that NSSI helps to ineffectively reduce negative affective states emerging from self-critical perfectionistic processes (Chester et al., 2015). Stronger experienced physical pain during NSSI in the “Severe/ Multimethod NSSI” subgroup could further confirm this link. In previous studies, it is revealed that physical pain endurance is predicted both by engaging in NSSI and higher self-critical beliefs (Hooley & St. Germain, 2014). This study reveals that the use of multiple severe NSSI methods co-occurs with stronger experienced pain in adolescents. Higher experienced pain could be a marker of self-punishment for adolescents.

In addition, the “Severe/Multimethod NSSI” class can be characterized with the worst mental health state. This group more likely included languishing adolescents who had low values on emotional, psychological, and social well-being. In community adolescent samples, proportion of languishing adolescents is usually around of 6% (Keyes, 2006). In this study, the “Severe/Multimethod NSSI” subgroup comprises languishing individuals more than five times greater in proportion (32%) when compared to nonclinical youth samples. This implies that nearly one third of these youth can be observed with low positive emotions and satisfaction with life, reduced self-acceptance, personal growth, purposes in life, autonomy, environmental mastery, and decreased positive relations with others, as well as poor social contribution and integration (Keyes, 2006). This incomplete mental health state is a major risk factor for the development of depressive symptoms (Keyes et al., 2010). Furthermore, internalizing symptoms and emotional problems are major risk factors for NSSI (Selby et al., 2012). Although a cross-sectional study is unable to detect causal links, it can be reasonably assumed that inadequate subjective well-being exacerbates not only depressive symptoms, but also unhealthy methods of emotion regulation, like NSSI. Moreover, severe NSSI may also have a detrimental effect on emotional, psychological, and social well-being. Previous research showed dampened positive emotional experiences among those who engaged in NSSI (Bresin, 2014). These findings imply that enhancing components of positive mental health can serve as a protective factor against severe and habituated NSSI.

The second and largest emerging class (61%) in the current study showed a constellation of NSSI methods represented by a medium level of banging or hitting self, and interfering with wound healing, combined with low probabilities of cutting, biting, carving, pinching, and scratching. Due to the lower number and less severity of the NSSI methods, this subgroup was labeled as “Mild/Moderate NSSI”. Hitting oneself may not necessarily be obviously visible and could go unnoticed by others in a social setting. Moreover, interfering with wound healing may prove to be an even more inconspicuous method of self-harm. These NSSI behaviors do not require instruments to be implemented, which may explain why a greater proportion of adolescents who engage in self-harm used these methods. It should be noted that the two emerging subgroups differ in terms of number of NSSI methods and intensity, as well as in intrapersonal motivation behind NSSI and positive mental health conditions. Therefore, further research is needed to identity influences that might prevent transitioning from “Mild/Moderate NSSI” to “Severe/ Multimethod NSSI” classes.

Quantitative differences could not be identified according to age, gender, perfectionistic standards and perfectionistic concerns, or interpersonal motivations behind NSSI between the two subgroups. In a prior adolescent NSSI LCA study, age was not associated with class membership (Somer et al., 2015), which may indicate that developmental transformation has little impact on existing NSSI latent class membership. Nevertheless, there is evidence that individual alteration, either decreasing or oscillating NSSI, in class membership could exist in a developmental context (Wang et al., 2017). Further longitudinal studies can shed light on whether, how and why adolescents shift across NSSI latent classes. Importantly, gender appears to have no impact on class membership, consistent with previous studies (Lloyd-Richardson et al., 2007). This result suggests that identifying as female is a risk factor for higher NSSI prevalence, but only when isolated from other factors (Bresin & Schoenleber, 2015). Furthermore, perfectionistic attitudes do not differ between the two NSSI severity classes. It appears that perfectionistic concerns are risk factors in general for engaging in NSSI independently of NSSI class membership (Gyori et al., 2021a).

The more at-risk group regarding NSSI, the “Severe/ Multimethod NSSI” class, is somewhat similar to the “Severe NSSI” (Case et al., 2020), the “Multimethod” (Singhal, 2021), or the “high rates of multiple forms of NSSI” (Somer et al., 2015) groups identified in previous LCA studies. Each of these groups showed higher psychological vulnerability. The “Mild/Moderate NSSI” group in this study is comparable with the “Experimental” (Klonsky & Olino, 2008), the “Mild/Experimental NSSI” (Case et al., 2020) or the “low endorsement in NSSI acts” (Somer et al., 2015) classes regarding the proportion and characteristics of NSSI methods. However, in contrast with previous studies, which primarily comprised emerging adult samples, in the current adolescent study, only two subgroups emerged. These differences in findings might be attributable to sample differences as well as to the focus on recently experienced NSSI instead of lifetime NSSI and the full suicidal self-injury spectrum.

Although findings from the present study increase our understanding of person-centered analysis of NSSI among adolescents, the study has limitations. First, the cross-sectional framework did not allow for detecting causal links or following developmental trajectories in NSSI subgroups. Furthermore, a community adolescent sample was explored, in which teens diagnosed with former or current mental illness could also be included. However, psychiatric history was not explored and therefore, could not be controlled. However, screening NSSI in generally healthy juvenile samples is important because of the growing prevalence of the phenomenon in general youth populations (Wester et al., 2018). Third, in this study, only secondary school students were involved. Considering the early onset of NSSI at approximately 12 years of age (Glenn & Klonsky, 2009), it is important to test whether the latent classes are replicable in early adolescence. Another limitation was the imbalanced gender ratio in the sample who engaged in NSSI in the previous month. Boys accounted for only one third of the initial and the current NSSI samples. Although the gender ratio in the total sample and in the NSSI sample were similar, future studies should recruit more representative samples based on gender to provide greater confidence in any detected differences in NSSI between boys and girls. Finally, adolescents completed study questionnaires in the school. Although trained investigators supervised this process, the stigma associated with NSSI may have resulted in socially desirable responding, which may affect the validity of our findings.

## Conclusion

Relatively little is known about the heterogeneity of NSSI subgroups in adolescents, in part because few studies have examined adolescent NSSI using a person-centered perspective. The present study used latent class analysis to explore different patterns of various NSSI acts, based on both the type and the severity of self-injury among youth. Among nonclinical adolescents who engaged in current (past month) NSSI, two robust classes emerged: a “Severe/Multimethod NSSI” subgroup with more impulsive engaging in multimethod self-injury and mainly for intrapersonal reasons, and a “Mild/Moderate subgroup,” who used fewer and less severe NSSI methods. Members of the former NSSI group had noticeably worse well-being. In school settings, identifying adolescents who are at risk for more severe or special conditions in self-injury can help to prevent the persistence of unhealthy coping behaviors from mid to late adolescence and from adolescence to young adulthood. Furthermore, identifying youth who engage in NSSI acts and tailoring effective interventions to their specific needs (e.g., analyzing and reducing intrapersonal motives behind NSSI, while enhancing emotional, psychological, and social well-being using positive psychology approaches) is particularly justified given the alarming increase in prevalence of these behaviors during a developmentally sensitive phase of life.

## Data Availability

The dataset generated and/or analyzed during the current study are not publicly available but are available from the corresponding author on reasonable request.
